# Potential mechanisms responsible for cardioprotective effects of sodium–glucose co-transporter 2 inhibitors

**DOI:** 10.1186/s12933-018-0745-5

**Published:** 2018-07-10

**Authors:** Charshawn Lahnwong, Siriporn C. Chattipakorn, Nipon Chattipakorn

**Affiliations:** 1 Cardiac Electrophysiology Research and Training Center, Faculty of Medicine, Chiang Mai University, Chiang Mai, Thailand; 2 Department of Pharmacology, Faculty of Medicine, Khon Kaen University, Khon Kaen, Thailand; 3 Department of Oral Biology and Diagnostic Sciences, Faculty of Dentistry, Chiang Mai University, Chiang Mai, 50200 Thailand; 4 Center of Excellence in Cardiac Electrophysiology Research, Chiang Mai University, Chiang Mai, 50200 Thailand; 5 Cardiac Electrophysiology Unit, Department of Physiology, Faculty of Medicine, Chiang Mai University, Chiang Mai, Thailand

**Keywords:** Sodium–glucose co-transporter 2 (SGLT-2) inhibitors, Heart, Diabetes mellitus

## Abstract

Diabetes mellitus currently affects over 350 million patients worldwide and is associated with many deaths from cardiovascular complications. Sodium–glucose co-transporter 2 (SGLT-2) inhibitors are a novel class of antidiabetic drugs with cardiovascular benefits beyond other antidiabetic drugs. In the EMPA-REG OUTCOME trial, empagliflozin significantly decreases the mortality rate from cardiovascular causes [38% relative risk reduction (RRR)], the mortality rate from all-causes (32% RRR) and the rate of heart failure hospitalization (35% RRR) in diabetic patients with established cardiovascular diseases. The possible mechanisms of SGLT-2 inhibitors are proposed to be systemic effects by hemodynamic and metabolic actions. However, the direct mechanisms are not fully understood. In this review, reports concerning the effects of SGLT-2 inhibitors in models of diabetic cardiomyopathy, heart failure and myocardial ischemia from in vitro, in vivo as well as clinical reports are comprehensively summarized and discussed. By current evidences, it may be concluded that the direct effects of SGLT-2 inhibitors are potentially mediated through their ability to reduce cardiac inflammation, oxidative stress, apoptosis, mitochondrial dysfunction and ionic dyshomeostasis.

## Background

Diabetes mellitus (DM) currently affects over 350 million patients globally [1]. The causes of death up to 80% in patients with type 2 DM (T2DM) are associated with cardiovascular diseases [2, 3]. Diabetic cardiomyopathy is a progressive disease which affects both cardiac structure and function in diabetic patients. These abnormalities include cardiac hypertrophy, cardiac apoptosis and necrosis, ventricular dilatation and interstitial fibrosis [4, 5] which consequently leads to both systolic and diastolic dysfunctions [6]. Metabolic disturbances, including hyperglycemia, insulin resistance and hyperlipidemia, play an important role in the diabetic cardiomyopathy process by triggering the renin–angiotensin–aldosterone system, altered lipid metabolism, inflammation, oxidative stress, mitochondrial dysfunction and endoplasmic reticulum (ER) stress [7]. Chronic exposure to these conditions make heart limited to physiological adaptation and repair capacity.

The sodium–glucose co-transporters (SGLT) are a family of active glucose transporter proteins with two major isoforms, SGLT-1 and SGLT-2 [8]. SGLT-1 expression found in the small intestine, liver, lung, kidney and heart, whereas SGLT-2 expression is predominantly found in the kidney [9]. SGLT-2 inhibitors are a novel class of antidiabetic drugs which produce glycosuric and natriuretic effects by inhibiting glucose and sodium reabsorption from the proximal convoluted tubules [10]. Some SGLT-2 inhibitors, including canagliflozin, dapagliflozin and empagliflozin, have been approved for their use in Europe and the USA [11]. Recently, SGLT-2 inhibitors have become the topic of interest due to the benefits in a cardiovascular outcome trial beyond other antidiabetic drugs. The EMPA-REG OUTCOME trial (2010–2015) showed the cardioprotective effect of empagliflozin by significantly lowering the rate of death from cardiovascular causes [38% relative risk reduction (RRR)], all-cause death (32% RRR) and heart failure hospitalization (HHF) (35% RRR) in T2DM patients with established cardiovascular diseases (CVD) [12]. These benefits of empagliflozin are expected to be class effects with SGLT-2 inhibitors. Several studies also supported this evidence [13–15] as summarized in Table 1. The CANVAS trial (2009–2017) showed canagliflozin significantly reduced the composite of cardiovascular-cause death, nonfatal myocardial infarction (MI) or nonfatal stroke [hazard ratio (HR) 0.86, 95% confidence interval (CI) 0.75–0.97] and HHF (HR 0.67, 95% CI 0.52–0.87) [13]. The CVD-REAL study which reported the cardiovascular effect of SGLT-2 inhibitors compared to other glucose-lowering drugs showed that SGLT-2 inhibitors could significantly decrease the rate of HHF (HR 0.49, 95% CI 0.41–0.57) and all-cause death (HR 0.61, 95% CI 0.51–0.73) [14]. Ongoing cardiovascular trials of SGLT-2 inhibitors include DECLARE-TIMI 58 (dapagliflozin, 2013–2019), VERTIS CV (ertugliflozin, 2013–2019) and RECEDE-CHF (empagliflozin, 2017–2019) may help to confirm this expectation [16–18].

**Table 1:** Summary of selected clinical studies reported on the effect of SGLT-2 inhibitors on cardiovascular outcomes

Model	n	Treatment	Major findings	Interpretation	References
T2DM + high CV risk	7020	RCTs (EMPA-REG OUTCOME trial); Empagliflozin (10 or 25 mg/days) vs. placebo/PO/3.1 years	↓ Cardiovascular-cause death (38% RRR) ↓ All-cause death (32% RRR) ↓ HHF (35% RRR) ↔ MI, stroke	Empagliflozin reduced the rate of HHF, death from cardiovascular and/or any causes in T2DM population at high risk for CV events	[12]
T2DM + high CV risk	10,142	RCTs (CANVAS trial); Canagliflozin (100 or 300 mg/days) vs. placebo/PO/3.6 years	↓ Composite of cardiovascular-cause death, nonfatal MI or nonfatal stroke (HR 0.86, 95% CI 0.75–0.97) ↓ HHF (HR 0.67, 95% CI 0.52–0.87) ↔ All-cause death, cardiovascular-cause death, nonfatal MI, nonfatal stroke	Canagliflozin reduced the rate of HHF and composite of death from cardiovascular causes, nonfatal MI or nonfatal stroke in T2DM population at high risk for CV events	[13]
T2DM	309,056	Retrospective observational (CVD-REAL study); SGLT-2 inhibitors vs. glucose-lowering drugs/> 1 year	↓ HHF (HR 0.49, 95% CI 0.41–0.57) ↓ All-cause death (HR 0.61, 95% CI 0.51–0.73)	SGLT-2 inhibitors reduced all-cause death and HHF compared with other glucose-lowering drugs in T2DM population	[14]
T2DM	14,697	Retrospective observational; SGLT-2 inhibitors vs. DPP-4 inhibitors/2 years	↓ HHF (HR 0.68, 95% CI 0.54–0.86)	SGLT-2 inhibitors reduced HHF compared with DPP-4 inhibitors in T2DM population	[15]

*SGLT-2* sodium–glucose co-transporter 2, *T2DM* type 2 diabetic mellitus, *CV* cardiovascular, *RCTs* randomized control trials, *vs* versus, *RRR* relative risk reduction, *HHF* hospitalization for heart failure, *MI* myocardial infarction, *HR* hazard ratio, *95% CI* 95% confidence interval, *DPP-4* dipeptidyl peptidase-4

Despite the cardiovascular benefits of SGLT-2 inhibitors, their biological mechanisms leading to cardioprotection are not fully understood. Possible mechanisms are clearly proposed to be systemic effects by hemodynamic actions via natriuresis and metabolic actions via glycosuria [19–23]. Natriuresis results in lowering plasma volume and blood pressure, which are subsequently decreasing cardiac preload and afterload [24–27]. This effect occurs without heart rate changes suggesting the lack of sympathetic stimulation [24, 28]. Empagliflozin also reduces arterial stiffness and vascular resistance in diabetic patients [28, 29]. In renal hemodynamics, empagliflozin attenuates albuminuria and hyperglycemia induced glomerular hyperfiltration, resulting in decreased intraglomerular hypertension [30–32]. The modulation of systemic and renal hemodynamics by SGLT2 inhibitors decreases cardiac workload and improves cardiac function. Glycosuria results in reducing plasma glucose concentration and subsequently decreasing glucotoxicity, a factor which leads to diabetic cardiomyopathy, as evidenced by the improvement of β cell function and insulin sensitivity [33, 34]. Interestingly, one of the benefits of SGLT-2 inhibitors beyond other hypoglycemic drugs is that they do not cause hypoglycemia, since they can enhance endogenous glucose production [33, 34]. The mechanism responsible for this benefit is the increase of plasma glucagon concentration by SGLT-2 inhibitors [33–35]. Dapagliflozin can directly stimulate pancreatic alpha cells for glucagon secretion [35]. Glucagon is known as the key hormone for hepatic glucose production [36, 37], enhances ketogenesis [38, 39] and improves cardiac contractility [40, 41]. Furthermore, SGLT-2 inhibitors can shift metabolism from carbohydrate to lipid [33, 42] and increase ketone body level in both animal and clinical studies [42–44]. The mechanisms responsible for that are potentially from compensatory mechanisms of glucose lowering drugs [45], reducing renal ketone body clearance [46] and enhancing ketone body production by glucagon action [33–35]. Ketone bodies are good energy source in myocardium especially in failing hearts [47, 48]. In addition, ketone bodies are associated with anti-arrhythmia and increasing mitochondrial biogenesis [49, 50]. This is known as the ketone body theory [51]. However, their benefits and mechanisms are still questionable because ketone bodies can precipitate diabetic ketoacidosis, a serious complication of diabetes [46]. Glycosuria also results in weight and fat mass reduction due to stimulation of lipid oxidation compensating for energy loss [52–54]. Both hemodynamic and metabolic actions of SGLT-2 inhibitors potentially work together for improving diabetic cardiomyopathy and finally resulting in cardioprotection as shown in the EMPA-REG OUTCOME trial. However, only systemic mechanisms may be not enough to explain much better cardiovascular benefits of SGLT-2 inhibitors when compared to other glucose-lowering drugs. Their direct cardiac mechanisms, even no SGLT-2 expression in the heart [9, 55, 56], may be the answers for that. Therefore, the objective of this review was to comprehensively summarize reports from in vitro, in vivo and clinical studies regarding the evidence of possible direct mechanisms responsible for cardioprotective effect of SGLT-2 inhibitors, which are independently from their systemic actions.

## Effects of SGLT-2 inhibitors on cardiac structure

SGLT-2 inhibitors have been shown to improve cardiac histopathologic changes in the diabetic cardiomyopathy models of mice and rats, the heart failure model of zebrafish embryos and also the myocardial ischemic model of rats. These reports are summarized in Table 2. In 2016, Kusaka et al. studied the effect of empagliflozin in genetic prediabetes/metabolic syndrome rat model [57]. After 10 weeks of treatment, empagliflozin significantly reduced left ventricular weight, cardiomyocyte size, cardiac interstitial fibrosis and cardiac interstitial macrophage infiltration. Several reports that studied the effect of empagliflozin in genetic diabetic mouse models also suggested the improvement of cardiac morphologic changes by decreasing the cardiomyocyte cross sectional area, interstitial collagen I and III depositions, interstitial fibrosis and interstitial macrophage infiltration [58, 59]. Lin et al. also reported that empagliflozin could attenuate pericoronary arterial fibrosis and coronary arterial thickening [58]. The reduction of cardiac fibrosis was shown to be due to the attenuation of the expression of pro-fibrotic signaling pathway, serum- and glucocorticoid-regulated kinase 1 (SGK1) and epithelial sodium channel (ENaC) [59]. In another genetic diabetic mouse model, empagliflozin was given for a total of 6 weeks [60]. Although there were no significant changes in left ventricular (LV) mass and histologic myocardial fibrosis, the treatment group showed the decrease of cardiac hypertrophy and remodeling markers. Empagliflozin was found to decrease the mRNA expression of hypertrophic fetal genes including atrial natriuretic peptide/factor and beta-myosin heavy chain. It also decreased the expression of proteins associated with mitogen-activated protein kinase pathways, including extracellular signal-regulated kinases, c-Jun NH2-terminal kinases and p38, which played an important role in the development of cardiac remodeling [61]. In streptozotocin-induced diabetic cardiomyopathy rats treated with empagliflozin for 8 weeks, the attenuation of disordered cell arrays and focal necrosis was observed in a dose-dependent manner [62]. Furthermore, Ye et al. showed that the treatment of dapagliflozin for 8 weeks decreased myocardial collagen-1 and collagen-3 mRNA levels and percentage of fibrosis in genetic diabetic mice [63]. These reports suggest that the improvement of diabetic cardiomyopathy morphology is potentially to be class effects of SGLT-2 inhibitors.

**Table 2:** Summary of the effects of SGLT-2 inhibitors on cardiac structure in animal models

Animal species	Model	Drug/dose/route	Major findings	Interpretation	References
Wistar rats	Streptozotocin-induced diabetic cardiomyopathy	Empagliflozin (30 or 10 mg/kg/days)/PO/8 weeks	↓ Disorganized cell arrays and focal necrosis in dose-dependent manner	Empagliflozin improved structural changes in the myocardium of streptozotocin-induced diabetic cardiomyopathy rats	[62]
SHR/NDmcr-cp(+/+) rats	Prediabetes/metabolic syndrome	0.03% empagliflozin/diet/10 weeks	↓ LV weight and cardiomyocyte size ↓ Interstitial fibrosis	Empagliflozin improved cardiac hypertrophy and interstitial fibrosis in genetic prediabetic metabolic syndrome rats	[57]
db/db mice	Diabetes/obesity (diastolic dysfunction and LVH)	Empagliflozin (10 mg/kg/days)/PO/5 weeks	↓ Cardiomyocyte cross sectional area ↓ Interstitial collagen I and III depositions ↓ SGK1 and ENaC expressions	Empagliflozin improved LVH and myocardial fibrosis in db/db mice	[59]
db/db mice	Diabetes/obesity (diastolic dysfunction and LVH)	0.03% empagliflozin/diet/10 weeks	↓ Cardiac interstitial and pericoronary arterial fibrosis ↓ Coronary arterial thickening	Empagliflozin attenuated cardiovascular remodeling in db/db mice	[58]
ob/ob mice	T2DM/obesity (LV diastolic dysfunctions)	Empagliflozin (10 mg/kg/days)/PO/6 weeks	↔ LV mass, myocardial fibrosis ↓ Anf, β-Mhc mRNA levels ↓ p-ERKs, p-JNKs and p-P38 expressions	Empagliflozin attenuated markers of cardiac hypertrophy and remodeling, but not altered cardiac fibrosis in ob/ob mice	[60]
BTBR ob/ob mice	T2DM	Dapagliflozin (1 mg/kg/days)/PO/8 weeks	↓ Collagen-1 and collagen-3 mRNA levels ↓ % fibrosis	Dapagliflozin attenuated cardiac fibrosis in BTBR ob/ob mice	[63]
Rats	High fat diet induced obese-insulin resistance for 4 weeks then I/R injury by LAD ligation	Dapagliflozin (1 mg/kg/days)/PO/4 weeks	↓ Infarct size	Dapagliflozin attenuated MI size in pre-diabetic rats with cardiac I/R injury	[64]
Wistar rats	MI by LAD ligation in rats	Dapagliflozin (0.1 mg/kg/days)/PO/Start after 1-day infarction for 4 weeks	↔ Infarct size ↓ Myofibroblast infiltration and cardiac fibrosis	Dapagliflozin attenuated myofibroblast infiltration during post-infarction remodeling in rats	[65]
cmlc2::GFP zebrafish embryos	Aristolochic acid induced heart failure	Empagliflozin (0.1, 10 μM)/media/3 days	↓ Morphologic changes in a concentration-dependent manner included unlooping defects, cardiac edema, and deformed cardiac chambers ↓ ANP, BNP signaling	Empagliflozin improved heart failure morphology and attenuated heart failure markers in zebrafish embryos	[66]

*SGLT-2* sodium–glucose co-transporter 2, *PO* per oral, *LV* left ventricular, *LVH* left ventricular hypertrophy, *SGK1* serum and glucocorticoid-inducible kinase 1, *ENaC* epithelial Na^+^ channel, *T2DM* type 2 diabetic mellitus, *Anf* atrial natriuretic peptide/factor, *β-Mhc* beta-myosin heavy chain, *p-ERKs* phosphorylated extracellular signal-regulated kinase, *p-JNKs* phosphorylated c-Jun NH2-terminal kinase, *I/R* ischemic/reperfusion, *LAD* left anterior descending artery, *MI* myocardial infarction

In addition to the diabetic cardiomyopathy model, SGLT-2 inhibitors were also tested in models of myocardial ischemia and heart failure. We recently demonstrated that by giving dapagliflozin for 4 weeks in high fat diet induced obese-insulin resistance rats and underwent acute ischemic/reperfusion (I/R) injury by left anterior descending artery (LAD) ligation, dapagliflozin could attenuate myocardial infarct size [64]. In chronic MI rat model, Lee et al. investigated the effect of dapagliflozin treatment beginning 1 day after LAD ligation and continued for 4 weeks [65]. They found that dapagliflozin did not alter the size of an infarction, however it could attenuate myofibroblast infiltration and cardiac fibrosis. In 2017, Shi et al. tested the effect of empagliflozin in aristolochic acid induced heart failure in zebrafish embryos [66]. Zebrafish embryos treated with aristolochic acid would develop cardiac hypertrophy, bradycardia and profound cardiac failure within 3 days of age [67]. Pretreatment with empagliflozin showed the improvement of histopathologic changes including unlooping defects, cardiac edema and deformed cardiac chambers in a concentration-dependent manner [66]. Furthermore, empagliflozin could attenuate the expression of heart failure markers including atrial natriuretic peptide and brain natriuretic peptide. In a very recent clinical trial, Januzzi et al. tested the effect of 2-year canagliflozin treatment in 666 elderly T2DM patients [68]. Compared to placebo, treatment with canagliflozin delayed the rise of heart failure biomarkers including serum N-terminal pro-brain natriuretic peptide and high-sensitivity troponin I.

## Effects of SGLT-2 inhibitors on cardiac function

SGLT-2 inhibitors have also been shown to improve cardiac function in diabetic cardiomyopathy models and myocardial ischemic models of mice and rats as summarized in Table 3. In genetic diabetic mice, 8-week treatment of dapagliflozin improved ejection fraction (EF) and fractional shortening [63]. It also attenuated the increase in end-systolic volume (ESV), end-diastolic volume (EDV), interventricular septal thickness in systole and diastole. Dapagliflozin also improved the E/A (early/late diastolic) ratio, EF, isovolumic relaxation time (IVRT), deceleration time (DT) and end diastolic wall thickness (EDWT) in a diabetic non-obese mouse model [69]. These reports indicated that dapagliflozin could improve both systolic and diastolic LV function in diabetic mice.

**Table 3:** Summary of the effects of SGLT-2 inhibitors on cardiovascular function in animal models

Animal species	Model	Drug/dose/route	Major findings	Interpretation	References
Wistar rats	Streptozotocin-induced diabetic cardiomyopathy	Empagliflozin (30 or 10 mg/kg/days)/PO/8 weeks	↑ ESP, + dp/dt and − dp/dt ↓ EDP	Empagliflozin improved LV function in streptozotocin-induced diabetic cardiomyopathy rats	[62]
SHR/NDmcr-cp (+/+) rats	Prediabetes/metabolic syndrome	0.03% empagliflozin/diet/7 weeks	↔ HR, SBP, DBP, locomotor activity, LF-SBP, sBRG and LF/HF ratio of PI	Empagliflozin did not have effect on heart rate, blood pressure, sympathetic activity, or baroreceptor function in genetic prediabetic metabolic syndrome rats	[57]
db/db mice	Diabetes/obesity (diastolic dysfunction and LVH)	Empagliflozin (10 mg/kg/days)/PO/5 weeks	↑ Septal wall motion ↓ CO, SV, LV filling pressure ↔ EF, FS	Empagliflozin improved septal wall motion and LV filling pressure in db/db mice	[59]
db/db mice	Diabetes/obesity (diastolic dysfunction and LVH)	0.03% empagliflozin/diet/10 weeks	↓ Impairment of vascular endothelium-dependent relaxation in thoracic aortas	Empagliflozin attenuated vascular dilating dysfunction in db/db mice	[58]
ob/ob mice	T2DM/obesity (LV diastolic dysfunctions)	Empagliflozin (10 mg/kg/days)/PO/6 weeks	↓ E wave, E wave deceleration time, *Tau*, EDPVR ↔ HR, EF, FS and ESPVR	Empagliflozin improved LV diastolic function but not systolic function in ob/ob mice	[60]
BTBR ob/ob mice	T2DM	Dapagliflozin (1 mg/kg/days)/PO/8 weeks	↓ ESV, EDV, IVSs, IVSd ↑ EF, FS ↔ HR	Dapagliflozin improved LV function in BTBR ob/ob mice	[63]
Seipin knockout (SKO) mice	Diabetic lipodystrophic cardiomyopathy	Dapagliflozin (1 mg/kg/days)/PO/8 weeks	↑ E/A ratio and EF ↓ IVRT, DT and EDWT ↔ EDV	Dapagliflozin improved both systolic and diastolic LV function in SKO mouse	[69]
Rats	High fat diet induced obese-insulin resistance for 4 weeks then I/R injury by LAD ligation	Dapagliflozin (1 mg/kg/days)/PO/4 weeks	*Before I/R injury* ↑ E/A ratio and EF ↓ HR, IVRT, DT and EDWT *During I/R injury* ↑ Time to 1st VT/VF onset ↓ Arrhythmia score ↑ Gap junction protein connexin 43 expression ↔ Number of VT/VF and VT/VF incidence ↑ ESP, SV, EF and SW ↓ HR, EDP, ESV and EDV	Dapagliflozin improved both systolic and diastolic LV function and heart rate variability in pre-diabetic rats Dapagliflozin prevented cardiac arrhythmia in pre-diabetic rats with cardiac I/R injury	[64]
Wistar rats	MI by LAD ligation in rats	Dapagliflozin (0.1 mg/kg/days)/PO/Start after 1-day infarction for 4 weeks	↑ Maximal rate of LV + dP/dt and − dP/dt	Dapagliflozin improved cardiac function during post-infarction remodeling in rats	[65]

*SGLT-2* sodium–glucose co-transporter 2, *PO* per oral, *ESP* end-systolic pressure, *EDP* end-diastolic pressure, + *dp/dt* maximal ascending rate of left ventricular pressure, − *dp/dt* maximal descending rate of left ventricular pressure, *LV* left ventricular, *HR* heart rate, *SBP* systolic blood pressure, *DBP* diastolic blood pressure, *LF* low frequency, *sBRG* spontaneous baroreceptor reflex gain, *HF* high frequency, *PI* pulse interval, *LVH* left ventricular hypertrophy, *CO* cardiac output, *SV* stroke volume, *EF* ejection fraction, *FS* fractional shortening, *T2DM* type 2 diabetic mellitus, *E wave* mitral inflow peak velocity, *Tau* time constant for isovolumic relaxation, *ESPVR* end-systolic pressure–volume relationship, *EDPVR* end-diastolic pressure–volume relationship, *ESV* end-systolic volume, *EDV* end-diastolic volume, *IVSs* interventricular septal thickness in systole, *IVSd* interventricular septal thickness in diastole, *E/A* early/late diastolic, *IVRT* isovolumic relaxation time, *DT* deceleration time, *EDWT* end diastolic wall thickness, *I/R* ischemic/reperfusion, *LAD* left anterior descending artery, *VT* ventricular tachycardia, *VF* ventricular fibrillation, *SW* stroke work, *MI* myocardial infarction

For empagliflozin, a number of reports showed its benefits preferred diastolic function to systolic function [57–60, 62]. In genetic diabetic mice, empagliflozin improved diastolic function as seen by increased septal wall motion and decreased LV filling pressure [59]. It also attenuated vascular dilating dysfunction by ameliorating the impairment of vascular endothelium-dependent relaxation in thoracic aortas [58]. Moreover, empagliflozin has been shown to improve LV diastolic function, both in relaxation and compliance, as evidenced by a decrease in E wave (mitral inflow peak velocity), E wave deceleration time, Tau (time constant for isovolumic relaxation) and end-diastolic pressure–volume relationship [60]. However, LV systolic function was not affected in this report. In a diabetic cardiomyopathy rat model, empagliflozin also improved LV function by the increase of end-systolic pressure (ESP), + dp/dt and − dp/dt (the maximal ascending rate and the maximal descending rate of left ventricular pressure, respectively) and the decrease of end-diastolic pressure (EDP) [62]. However, in the prediabetic/metabolic syndrome rat model, 10 weeks of empagliflozin treatment, which attenuated LV weight and cardiac interstitial fibrosis, did not significantly improve heart rate, blood pressure, sympathetic activity or baroreceptor function [57].

Electrophysiologically, two recent clinical studies reported the effect of SGLT-2 inhibitors on electrocardiographic parameters in patients with T2DM [68, 70]. Sato et al. retrospectively analyzed changes in indices of ventricular repolarization before and after 0.66-year treatment with SGLT-2 in 46 people with T2DM [70]. They found the heart rate and QTc interval were not changed, but QTc dispersion was significantly decreased, suggesting that SGLT-2 inhibitors could reverse ventricular repolarization heterogeneity in T2DM patients. However, inconsistent findings exist. Januzzi et al. demonstrated the negative results from a randomized control trial which tested the effect of a 2-year canagliflozin treatment compared to placebo in 666 elderly T2DM patients [68]. They found that canagliflozin did not change any electrocardiographic parameters including PR interval, QRS interval, QT/QTc or RR intervals. Therefore, more clinical trials are required to assess the effects of SGLT-2 inhibitors on the cardiovascular function.

In addition to the diabetic cardiomyopathy models, SGLT-2 inhibitors also improved cardiac function in models of myocardial ischemia. In 2018, Tanajak et al. tested the dapagliflozin effect in obese-insulin resistance rats with I/R injury [64]. Before I/R injury operation, 4-week dapagliflozin treatment already showed the improvement of E/A ratio, EF, IVRT, DT and EDWT which referred to both systolic and diastolic LV function. Furthermore, during I/R injury, rats treated with dapagliflozin gave the increase of time to the first ventricular tachycardia/fibrillation onset, the increase of gap junction protein connexin 43 expression, the decrease of arrhythmia score and the improvement of EF, stroke volume, ESP, EDP, ESV and EDV. Similar results were found in Wistar rats with MI treated with dapagliflozin for 4 weeks in which the improvement of cardiac function as evidenced by increasing the maximal rate of LV + dP/dt and − dP/dt were observed [65]. Based on the EMPA-REG OUTCOME trial, there were no significant differences in the rates of MI between the placebo and treatment groups [12]. However, as evidenced from animal studies, SGLT-2 inhibitors could potentially exert beneficial effects to decrease the severity of MI both structural and function. Future clinical studies are needed to warrant these findings from basic reports.

## Potential mechanisms of SGLT-2 inhibitors responsible for cardioprotection

### SGLT-2 inhibitors on cardiac inflammation

Cardiac inflammation is one of the mechanisms that leads to diabetic cardiomyopathy in diabetic patients [71–73]. Evidence shows that SGLT-2 inhibitors, together with systemic effects, could directly decrease cardiac inflammation. These effects of SGLT-2 inhibitors on cardiac inflammation are summarized in Table 4. In the genetic prediabetes/metabolic syndrome rat model, 10 weeks of empagliflozin treatment significantly reduced cardiac interstitial macrophage infiltration [57]. Empagliflozin could also attenuate cardiac macrophage infiltration by decreasing cell numbers in the genetic diabetes/obesity mouse model [58]. Lee et al. also reported the improvement of cardiac inflammation in Wistar rats with acute phase of MI treated with dapagliflozin for 2 days [65]. They revealed that dapagliflozin decreased inflammatory cytokines mRNA levels including IL-1β and IL-6, increased anti-inflammatory cytokine mRNA levels including IL-10, and also increased the M2/M1 phenotype macrophage ratio. Since M1 is pro-inflammatory phenotype of macrophage, whereas M2 is anti-inflammatory one [74], these findings indicated that dapagliflozin promotes macrophage polarization toward an anti-inflammatory phenotype. Furthermore, empagliflozin could attenuate the myocardial expression of pro-inflammatory genes including cyclooxygenase-2 and interleukin-1β (IL-1β) in a heart failure model of zebrafish embryos [66]. All of these findings suggest that empagliflozin can reduce cardiac inflammation in diabetic cardiomyopathy, myocardial ischemia and heart failure models. However, we cannot conclude whether these benefits are from systemic and/or direct cardiac effects. More in vitro studies are required to explore its direct role independently from systemic one.

**Table 4:** Summary of the effects of SGLT-2 inhibitors on cardiac inflammation in animal models

Animal species	Model	Drug/dose/route	Major findings	Interpretation	References
SHR/NDmcr-cp (+/+) rats	Prediabetes/metabolic syndrome	0.03% empagliflozin/diet/10 weeks	↓ Cardiac interstitial macrophage infiltration	Empagliflozin attenuated cardiac macrophage infiltration in genetic prediabetic metabolic syndrome rats	[57]
db/db mice	Diabetes/obesity (diastolic dysfunction and LVH)	0.03% empagliflozin/diet/10 weeks	↓ Cardiac macrophage infiltration	Empagliflozin attenuated cardiac macrophage infiltration in db/db mice	[58]
BTBR ob/ob mice	T2DM	Dapagliflozin (1 mg/kg/days)/PO/8 weeks	↓ NALP-3, ASC, caspase-1, IL-1β, IL-6 and TNFα expressions	Dapagliflozin attenuated NLRP3 inflammasome and inflammatory markers in BTBR ob/ob mice	[63]
BTBR ob/ob mice	In vitro cardiofibroblast culture	Dapagliflozin (0.1–0.5 μM)/media/16 h	↓ NALP-3, ASC, caspase-1 and IL-1β expressions in dose dependent manner	Dapagliflozin attenuated NLRP3 inflammasome and inflammatory markers in cardiofibroblasts of BTBR ob/ob mice These effects are independent from SGLT-2 and glucose reducing effects of dapagliflozin	[63]
Wistar rats	MI by LAD ligation in rats	Dapagliflozin (0.1 mg/kg/days)/PO/Start after 1-day infarction for 2 days	↑ M2/M1 phenotype macrophage ratio and IL-10 mRNA level ↓ IL-1β and IL-6 mRNA levels	Dapagliflozin promoted macrophages toward anti-inflammatory phenotype, increased anti-inflammatory cytokine and attenuated inflammatory cytokines in rats with acute stage of MI	[65]
cmlc2::GFP zebrafish embryos	Aristolochic acid induced heart failure	Empagliflozin (0.1, 10 μM)/media/3 days	↓ Cox-2, IL-1β expressions	Empagliflozin attenuated pro-inflammatory genes in heart failure zebrafish model	[66]

*SGLT-2* sodium–glucose co-transporter 2, *LVH* left ventricular hypertrophy, *T2DM* type 2 diabetic mellitus, *NALP-3* NACHT, LRR and PYD domains-containing protein 3, *ASC* apoptosis-associated speck-like protein containing a caspase recruitment domain, *IL* interleukin, *TNFα* tumor necrosis factor α, *NLRP3* nucleotide-binding oligomerization domain-like receptor 3, *MI* myocardial infarction, *LAD* left anterior descending artery, *Cox-2* cyclooxygenase-2

Interestingly, a recent study revealed the direct mechanism of SGLT-2 inhibitors on cardiac inflammation reduction through the reduction of cardiac nucleotide-binding oligomerization domain-like receptor 3 (NLRP3) inflammasome [63]. The NLRP3 inflammasome is an interleukin-1β family cytokine-activating multi-protein signaling complex upregulated in the heart and associated with cardiac inflammation in T2DM, which leads to subsequent diabetic cardiomyopathy [75–79]. NACHT, LRR and PYD domains-containing protein 3 (NALP-3), the protein encoded by the NLRP3 gene, together with apoptosis-associated speck-like protein containing a caspase recruitment domain (ASC) form a protein complex activating caspase-1, which subsequently leads to stimulating the production of pro-inflammatory cytokines [72, 73]. Ye et al. tested the effect of 8-week dapagliflozin treatment on cardiac inflammation in genetic diabetic mice and found that dapagliflozin decreased the levels of myocardial mRNA associated with NLRP3 inflammasome and pro-inflammatory cytokines including NALP-3, ASC, caspase-1, IL-1β, IL-6 and TNFα [63]. To rule out systemic effects, they further performed in vitro experiment by incubating mouse cardiofibroblasts in media containing dapagliflozin for 16 h. Interestingly, dapagliflozin also attenuated NALP-3, ASC, caspase-1 and IL-1β mRNA levels in a dose-dependent manner [63]. Since SGLT-2 does not exist in cardiac tissue [9, 55, 56], these results suggested that these effects are unrelated from SGLT-2 and glucose reducing effects of dapagliflozin.

### SGLT-2 inhibitors on cardiac oxidative stress

Oxidative stress plays an important role in the pathogenesis of cardiac hypertrophy and remodeling [80–82]. SGLT-2 inhibitors have been shown to act as antioxidants by decreasing cardiac oxidative stress, independently from glucose lowering effects, as evidence summarized in Table 5. In genetic prediabetes/metabolic syndrome rat model, 10 weeks of empagliflozin treatment significantly reduced superoxide levels in cardiac tissues [57]. This report showed the reduction of cardiac hypertrophy and interstitial fibrosis although no blood pressure reduction or improvement of cardiac autonomic dysfunction. Therefore, these cardioprotective effects have been attributed to the lowering of cardiac oxidative stress and inflammation. In a genetic diabetic mouse model, 10-week empagliflozin treatment could decrease cardiac and aortic superoxide levels [58]. In a diabetes/obesity mouse model, Habibi et al. showed empagliflozin (10 mg/kg/days) did not alter the levels of cardiac nitrotyrosine, advanced glycation end products (AGEs) and receptors for AGEs (RAGEs) [59]. In aortic tissues of diabetic cardiomyopathy rats, empagliflozin treatment with high dose (30 mg/kg/days) significantly decreased AGEs and RAGEs levels but was unchanged in the low dose (10 mg/kg/days) treatment group [83]. Therefore, a high dose of empagliflozin treatment is required for oxidative stress reduction.

**Table 5:** Summary of the effects of SGLT-2 inhibitors on cardiac oxidative stress in animal models

Animal species	Model	Drug/dose/route	Major findings	Interpretation	References
SHR/NDmcr-cp (+/+) rats	Prediabetes/metabolic syndrome	0.03% empagliflozin/diet/10 weeks	↓ Cardiac superoxide level	Empagliflozin attenuated cardiac oxidative stress in genetic prediabetic metabolic syndrome rats	[57]
db/db mice	Diabetes/obesity (diastolic dysfunction and LVH)	Empagliflozin (10 mg/kg/days)/PO/5 weeks	↔ Cardiac nitrotyrosine level ↔ AGEs and RAGEs expressions	Empagliflozin had no effect on myocardial oxidative/nitrosative stress in db/db mice	[59]
db/db mice	Diabetes/obesity (diastolic dysfunction and LVH)	0.03% empagliflozin/diet/10 weeks	↓ Cardiac and aortic superoxide levels	Empagliflozin attenuated cardiovascular oxidative stress in db/db mice	[58]
Rats	High fat diet induced obese-insulin resistance for 4 weeks then I/R injury by LAD ligation	Dapagliflozin (1 mg/kg/days)/PO/4 weeks	↓ Malondialdehyde level in ischemic area	Dapagliflozin attenuated cardiovascular oxidative stress in pre-diabetic rats with cardiac I/R injury	[64]
Wistar rats	MI by LAD ligation in rats	Dapagliflozin (0.1 mg/kg/days)/PO/Start after 1-day infarction for 2 days	↓ Cardiac superoxide and nitrotyrosine levels ↑ STAT3 activation	Dapagliflozin attenuated cardiac oxidative/nitrosative stress and increased RONS-dependent STAT3-mediated pathway in rats with acute stage of MI	[65]
Wistar rats	Ex vivo Isolated hearts after 3-day infarction by LAD ligation	Dapagliflozin (10 μM)/media/1 h	↑ STAT3 activation ↑ IL-10 protein level	Dapagliflozin activated RONS-dependent STAT3-mediated pathway independently from its SGLT-2 and glucose lowering effects	[65]

*SGLT-2* sodium–glucose co-transporter 2, *LVH* left ventricular hypertrophy, *PO* per oral, *AGEs* advanced glycation end products, *RAGEs* receptors for AGEs, *I/R* ischemic/reperfusion, *MI* myocardial infarction, *LAD* left anterior descending artery, *STAT3* signal transducer and activator of transcription 3, *RONS* reactive oxygen and nitrogen species

For a model of acute myocardial ischemia, we previously demonstrated that dapagliflozin attenuated malondialdehyde in the cardiac ischemic area of obese-insulin resistant rats with I/R injury [64]. In a chronic MI model of rats, Lee et al. showed that dapagliflozin acted as an antioxidant and mediated M2 macrophage polarization through the signal transducer and activator of the transcription 3 (STAT3) mediated pathway [65]. Antioxidants, the reactive oxygen and nitrogen species (RONS) scavengers, are known to increase STAT3 activity which subsequently plays an action in M2 macrophage polarization upregulation, resulting in a decrease of cardiac inflammation [84, 85]. Moreover, myocardial ischemic rats fed with dapagliflozin for 2 days demonstrated the attenuation of superoxide and nitrotyrosine levels in cardiac tissues [65]. Dapagliflozin also increased STAT3 activity and stimulated macrophages toward an anti-inflammatory phenotype through STAT3 signaling [65]. In an ex vivo experiment using isolated hearts after 3-day infarction treated with dapagliflozin for 1 h, dapagliflozin-treated hearts still had increased STAT3 activity and IL-10 protein levels [65]. This evidence supports the role of dapagliflozin in MI as antioxidant and inflammatory modulators through direct RONS-dependent STAT3 signaling, independently from its SGLT-2 and glucose lowering effects.

### SGLT-2 inhibitors on cardiac apoptosis

Cardiac apoptosis has been shown to be responsible for cardiomyocyte death during MI and heart failure [86–88]. Accumulating reports have demonstrated that SGLT-2 inhibitors could decrease cardiac apoptosis in diabetic models and myocardial ischemic model of mice and rats as summarized in Table 6. In genetic diabetic mouse model, dapagliflozin treatment for 8 weeks significantly attenuated apoptotic cells in the left ventricle [63]. Consistently, diabetic cardiomyopathy rats treated with empagliflozin for 8 weeks had a decreased level of apoptotic cardiomyocytes [62]. This effect appeared in a dose-dependent manner since the number of apoptotic cells in high dose treated group (30 mg/kg/days) was significant lower than that in a low dose (10 mg/kg/days) treated group. In that report [62], it has been proposed that empagliflozin protected against cardiomyocyte apoptosis by suppression of the endoplasmic reticulum stress (ERS) pathway.

**Table 6:** Summary of the effects of SGLT-2 inhibitors on cardiac apoptosis in animal models

Animal species	Model	Drug/dose/route	Major findings	Interpretation	References
Wistar rats	Streptozotocin-induced diabetic cardiomyopathy	Empagliflozin (30 or 10 mg/kg/days)/PO/8 weeks	↓ Apoptotic cells ↓ GRP78, CHOP protein expression ↓ Caspase-12 activity ↓ ATF4, TRAF2, and XBP1 mRNA All effects are in dose-dependent manner	Empagliflozin, in dose-dependent manner, attenuated cardiomyocyte apoptosis by suppressing the endoplasmic reticulum stress pathway in streptozotocin-induced diabetic cardiomyopathy rats	[62]
ob/ob mice	T2DM/obesity (LV diastolic dysfunctions)	Empagliflozin (10 mg/kg/days)/PO/6 weeks	↔ Bcl2 and Bax levels	Empagliflozin had no effect on apoptotic protein expressions in ob/ob mice	[60]
BTBR ob/ob mice	T2DM	Dapagliflozin (1 mg/kg/days)/PO/8 weeks	↓ Apoptotic cells	Dapagliflozin attenuated cardiomyocyte apoptosis in BTBR ob/ob mice	[63]
Rats	High fat diet induced obese-insulin resistance for 4 weeks then I/R injury by LAD ligation	Dapagliflozin (1 mg/kg/days)/PO/4 weeks	↓ Bax/Bcl-2 ratio ↓ Cleavage caspase 3 level	Dapagliflozin attenuated apoptotic protein expressions in pre-diabetic rats with cardiac I/R injury	[64]

*SGLT-2* sodium–glucose co-transporter 2, *PO* per oral, *GRP78* glucose-regulated protein 78, *CHOP* CCAAT-enhancer-binding protein homologous protein, *ATF4* activating transcription factor 4, *TRAF2* tumor necrosis factor receptor-associated factor 2, *XBP1* X-box binding protein 1, *T2DM* type 2 diabetic mellitus, *LV* left ventricular, *Bcl-2* B-cell chronic lymphocytic leukemia/lymphoma-2, *Bax* Bcl-2-associated X, *I/R* ischemic/reperfusion, *LAD* left anterior descending artery

ERS is one of the pathological conditions in the diabetic cardiomyopathy which activated reactive oxygen species (ROS)-mediated cell apoptosis [89]. ERS can be stimulated by situations such as hyperglycemia, hypoxia, and ROS exposure, which results in abnormal protein folding and maturation leading to apoptosis [90]. Inhibitions of ERS could attenuate myocardial apoptosis and diabetic cardiomyopathy development in streptozotocin-induced diabetic rats [91, 92]. In response to ERS, glucose-regulated protein 78 (GRP78), a major ER chaperone protein, is activated and plays a vital role in detecting the anomalous proteins [93]. Once ERS occurred, caspase-12-mediated apoptosis, which is a unique apoptosis pathway of ER, is activated [94, 95]. Then, CCAAT-enhancer-binding protein homologous protein (CHOP), a subsequent protein of the apoptotic pathway, can stimulate the caspase protein in the cytosol, leading to apoptosis [96]. CHOP can be activated by the over-transcription of activating transcription factor 4 (ATF4), tumor necrosis factor receptor-associated factor 2 (TRAF2) and X-box binding protein 1 (XBP1) [97, 98]. It has been shown that empagliflozin decreased all of mRNA and protein expressions associated with ERS including GRP78, CHOP, Caspase-12, ATF4, TRAF2 and XBP1 [62]. Interestingly, these beneficial effects are in a dose-dependent manner. Thus, another role of empagliflozin in protection against diabetic cardiomyopathy is by attenuating cardiomyocyte apoptosis through inactivating the ERS pathway. However, inconsistent findings exist. A report by Hammoudi and colleagues demonstrated that empagliflozin (10 mg/kg/days) did not alter the protein expression of the antiapoptotic molecule Bcl-2 and the pro-apoptotic protein Bax in diabetic mice [60]. These inconsistent findings could be due to different doses of drug used; empagliflozin treatment at a low dose might not show an improvement [59, 83]. Future studies using a higher dose of empagliflozin treatment should be investigated in in vivo to explore its role on an apoptotic protein expression in diabetic cardiomyopathy model.

In myocardial I/R injury model, it has been shown that treatment with dapagliflozin for 4 weeks in obese-insulin resistance rats attenuated Bax/Bcl-2 ratio and cleaved caspase 3 level when these rats underwent I/R injury [64]. By current evidences, SGLT-2 inhibitors seem to attenuate apoptotic myocardial cells in diabetic cardiomyopathy and MI, however more studies are required to explore their roles on cardiac apoptosis.

### SGLT-2 inhibitors on cardiac mitochondrial function

Mitochondria are important to maintain physiological cardiac function due to their roles in energy production, calcium homeostasis and ROS production [99]. Mitochondrial dysfunction is found to be associated with the pathological progression of diabetic cardiomyopathy [100]. Impaired mitochondrial function and dynamics are observed in diabetic patients and leads to myocardial contractile dysfunction [101]. Several reports showed the attenuation of mitochondrial dysfunction by SGLT-2 inhibitor treatment [59, 64].

In a genetic diabetic mouse model, empagliflozin has been shown to attenuate ultrastructural anomalies of inter-myofibrillar mitochondria including disorganized appearance of sarcomeres, reduced matrix electron density, loss of cristae and mitochondrial fragmentation [59]. In obese-insulin resistant rats treated with dapagliflozin for 4 weeks before undergoing cardiac I/R injury, dapagliflozin attenuated the increase of mitochondrial ROS production, depolarization and mitochondrial swelling [64]. The mitochondrial morphology was also improved by attenuating mitochondrial fragmentation, loss of cristae and fusion of cristae. For mitochondrial biogenesis, dapagliflozin increased the protein expressions of peroxisome proliferator-activated receptor gamma coactivator 1-alpha (PGC1-α) and carnitine palmitoyltransferase 1 (CPT1), which were essential proteins for the regulation of cardiac mitochondrial fatty acid oxidation [102, 103]. The expression of complex I of the electron transport chain was also increased by dapagliflozin treatment, suggesting its role in restoring the reduction of cardiac energy metabolism during cardiac I/R injury [64].

It is known that mitochondria are dynamic organelles with the balance of continual fission (division) and fusion (joining) [104]. These cycles maintain functional mitochondria by removing damaged mitochondria and facilitating apoptosis when cells are exposed to stress [105]. Dynamin-related protein 1 (DRP1) plays role in constriction of the membrane during fission whereas mitofusin 2 (MFN2) and optic atrophy 1 (OPA1) support fusion of the outer and inner membranes, respectively [100]. It has been shown that an inhibition of cardiac mitochondrial fission could protect the heart during myocardial I/R injury and cardiac arrest [106, 107]. Recently, we have demonstrated that a 4-week treatment with dapagliflozin attenuated the increase of cardiac mitochondrial fission and the decrease of mitochondrial fusion as evidenced by decreased DRP1 and increased MFN2 and OPA1 expressions in obese-insulin resistant rats undergoing cardiac I/R injury [64]. The improvement of mitochondrial dynamics observed in this study, together with the improvement of mitochondrial function, morphology, ROS production, biogenesis and protein expressions could be the mechanisms responsible for smaller infarct size with dapagliflozin treatment compared to placebo. However, these benefits are still unclear whether they are from direct cardiac and/or systemic effects. More studies are needed to explore its direct role on the heart.

### SGLT-2 inhibitors on cardiac ionic homeostasis

Cardiac Ca^2+^ and Na^+^ homeostasis plays an important role in maintaining physiologic cardiac function, including rhythm and contraction [108, 109]. Intracellular Na^+^ and Ca^2+^ loading has been observed in diabetic hearts, particularly in heart failure [110–112]. Reduction of myocardial intracellular Na^+^ concentration by inhibition of Na^+^/Ca^2+^ or Na^+^/H^+^ exchangers improves heart failure and cardiac hypertrophy [113–115]. SGLT-2 inhibitors have been shown to improve cardiac Na^+^ and Ca^2+^, as evidence summarized in Table 7. Baartscheer and colleagues demonstrated the direct myocardial effects of empagliflozin on Na^+^ and Ca^2+^ concentration alteration, independent from SGLT-2 activity [116]. In isolated ventricular myocytes from rabbits and rats incubated with empagliflozin for 3 h, empagliflozin decreased cytoplasmic Na^+^ ([Na^+^]_c_) and Ca^2+^ ([Ca^2+^]_c_) and also increased mitochondrial Ca^2+^ concentration ([Ca^2+^]_m_). These effects were similar to the effect of Na^+^/H^+^ exchange (NHE) inhibitor [110]. These findings were confirmed in cells pre-treated with cariporide, a strong NHE inhibitor, in which the results showed that empagliflozin had very little effect on the [Na^+^]_c_ of these cells. Therefore, despite no SGLT-2 expression in the heart [9, 55, 56], empagliflozin could have cardiac effects by decreasing myocardial [Na^+^]_c_ and [Ca^2+^]_c_ and increasing [Ca^2+^]_m_ through the inhibition of NHE directly. Moreover, Liu and O’Rourke demonstrated that high [Na^+^]_c_ could cause low [Ca^2+^]_m_ through its efflux via mitochondrial Na^+^/Ca^2+^ exchanger (NCX) [117]. Increasing [Ca^2+^]_m_, could prevent sudden death in a swine heart failure model [113]. Taken together, SGLT-2 inhibitors directly inhibited myocardial NHE and consequently decreased the cytoplasmic Na^+^ level, leading to increased mitochondrial Ca^2+^ level and decreased cytoplasmic Ca^2+^ level through mitochondrial NCX activity. In addition, it has been shown that empagliflozin improved LV diastolic function by increasing sarcoplasmic endoplasmic reticulum Ca^2+^-ATPase (SERCA2a) activity in genetic diabetic mice [60]. Consistent with this report, Joubert et al. also showed that dapagliflozin increased SERCA2a function in a diabetic lipodystrophic mouse model [69]. SERCA2a is an important calcium handling protein which regulates cardiac contractility via Ca^2+^ reuptake into sarcoplasmic reticulum [118]. Decreased SERCA2a activity can cause abnormal Ca^2+^ handling and a contractile state, leading to cardiac contractile dysfunction [119]. All of these findings indicate that the improvement of cardiac calcium handling of SGLT-2 inhibitors through these mechanisms could be responsible for the protective effect in heart failure observed in the EMPA-REG OUTCOME trial.

**Table 7:** Summary of the effects of SGLT-2 inhibitors on cardiac ionic homeostasis in animal models

Animal species	Model	Drug/dose/route	Major findings	Interpretation	References
Rabbits and rats	In vitro isolated ventricular myocytes	Empagliflozin (1 μmol/l)/media/3 h	↓ Myocardial [Na^+^]_c_ and [Ca^2+^]_c_ ↑ Myocardial [Ca^2+^]_m_ These effects were strongly reduced after pre-treated with NHE inhibitor	Empagliflozin has cardiac effects by decreasing myocardial cytoplasmic [Na^+^]_c_ and [Ca^2+^]_c_ and increasing [Ca^2+^]_m_ through inhibition of NHE directly	[116]
ob/ob mice	T2DM/obesity (LV diastolic dysfunctions)	Empagliflozin (10 mg/kg/days)/PO/6 weeks	↑ SERCA2a/PLN ratio, PLN phosphorylation	Empagliflozin enhances SERCA2a activity leading to improve cardiac contractile dysfunction in ob/ob mice	[60]
Seipin knockout (SKO) mice	Diabetic lipodystrophic cardiomyopathy	Dapagliflozin (1 mg/kg/days)/PO/8 weeks	↑ SERCA2a/PLN ratio	Dapagliflozin enhances SERCA2a activity leading to improve cardiac contractile dysfunction in SKO mice	[69]

*[Na*^*+*^*]*_*c*_ cytoplasmic Na^+^ concentration, *[Ca*^*2+*^*]*_*c*_ cytoplasmic Ca^2+^ concentration, *[Ca*^*2+*^*]*_*m*_ mitochondrial Ca^2+^ concentration, *NHE* Na^+^/H^+^ exchange, *T2DM* type 2 diabetic mellitus, *LV* left ventricular, *PO* per oral, *SERCA2a* sarcoplasmic endoplasmic reticulum calcium (Ca^2+^) ATPase, *PLN* phospholamban

## Conclusions and perspectives

**Fig. 1:**
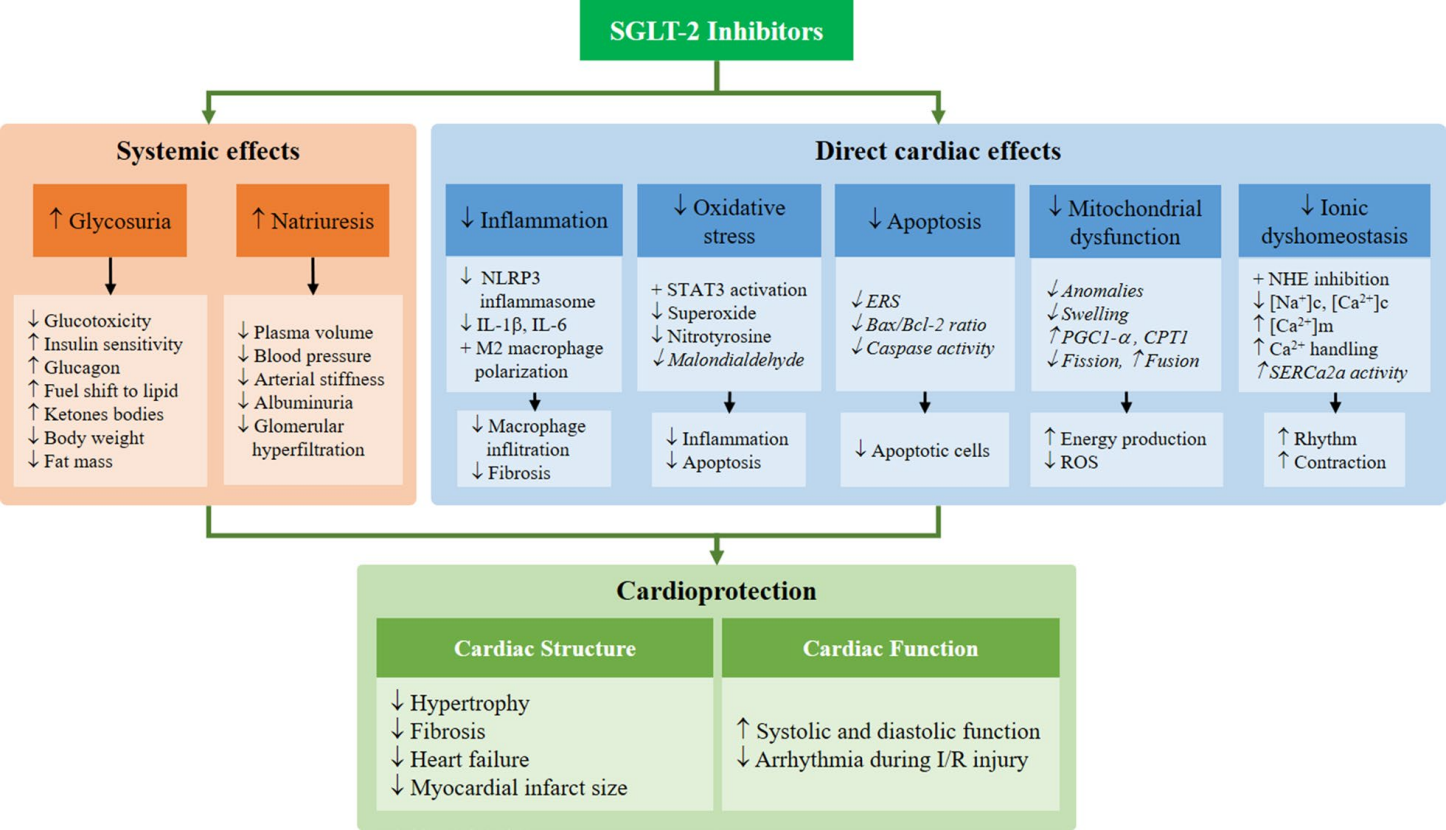
Potential mechanisms responsible for cardioprotective effect of SGLT-2 Inhibitors. From current evidence from both in vitro or ex vivo experiments, SGLT-2 inhibitors have been demonstrated that they could have direct cardiac effects on inflammation [63], oxidative stress [65], and ionic dyshomeostasis [116]. Although the effects of SGLT-2 inhibitors on the attenuation of apoptosis and mitochondrial dysfunction could be direct cardiac effects [59, 62, 64], they have not been proved by either in vitro or ex vivo experiments. Italics indicate the mechanisms have not been proved by either in vitro or ex vivo experiments. *SGLT*-*2* sodium–glucose co-transporter 2, *NLRP3* nucleotide-binding oligomerization domain-like receptor 3, *IL* interleukin, *STAT3* signal transducer and activator of transcription 3, *ERS* endoplasmic reticulum stress, *Bcl-2* B cell chronic lymphocytic leukemia/lymphoma-2, *Bax* Bcl-2-associated X, *PGC1-α* peroxisome proliferator-activated receptor gamma coactivator 1-alpha, *CPT1* carnitine palmitoyltransferase 1, *ROS* reactive oxygen species, *NHE* Na^+^/H^+^ exchange, *[Na*^*+*^*]*_*c*_ cytoplasmic Na^+^ concentration, *[Ca*^*2+*^*]*_*c*_ cytoplasmic Ca^2+^ concentration, *[Ca*^*2+*^*]*_*m*_ mitochondrial Ca^2+^ concentration, *SERCA2a* sarcoplasmic endoplasmic reticulum Ca^2+^-ATPase, *I/R* ischemic/reperfusion

The cardioprotection of SGLT-2 inhibitors has been demonstrated in models of diabetic cardiomyopathy, heart failure and myocardial ischemia. They are seen to be effective by improving cardiac morphologic changes including cardiac hypertrophy, interstitial fibrosis, heart failure and myocardial infarct size. They also improve both systolic and diastolic LV function in diabetic cardiomyopathy and prevent cardiac arrhythmia in cardiac I/R injury. Potential mechanisms responsible for the cardioprotective effects of SGLT-2 inhibitors are through direct and systemic effects which are summarized in Fig. 1. Their systemic effects are modulated by hemodynamic actions via natriuresis and metabolic actions via glycosuria. The direct effects of SGLT-2 inhibitors could potentially mediate through their abilities to attenuate cardiac inflammation, oxidative stress, apoptosis, mitochondrial dysfunction and ionic dyshomeostasis.

Focus on The EMPA-REG OUTCOME trial, empagliflozin decreased the rate of death from cardiovascular causes and HHF in T2DM patients with established CVD [12]. The mechanisms of SGLT-2 inhibitors responsible for these benefits could be due to their systemic as well as direct cardiac effects. SGLT-2 inhibitors can modify risk factors of major cardiovascular events including diabetes and hypertension via lowering blood glucose and blood pressure, respectively. They also directly attenuate cardiac inflammation, oxidative stress [63, 65] which lead to improving both cardiac structure and function, and finally result in decreased mortality rate from cardiovascular causes. For reducing HHF, natriuretic effect of SGLT-2 inhibitors results in lowering plasma volume and blood pressure, which are subsequently decreasing cardiac preload and afterload [24–26]. SGLT-2 inhibitors also directly improve cardiac calcium handling via inhibiting myocardial NHE which subsequently decrease intracellular Na^+^ and Ca^2+^ loading mostly found in heart failure [116]. Therefore, cardiac contractility and cardiac output could be improved in heart failure patients as observed in clinical trials [12–15].

Despite these growing number of reports, the cardioprotective effects in some respects such as cardiac apoptosis and mitochondrial dysfunctions are still unclear whether they are the direct effects or systemic effects of SGLT-2 inhibitors. More studies of heart failure and myocardial ischemic models are required to investigate the roles of SGLT-2 inhibitors on the heart. Although SGLT-2 does not exist in the myocardium [9, 55, 56], Park et al. reported that endothelial cells of porcine coronary artery exposed to high glucose upregulated SGLT-2 expression despite no expression in normal condition [120]. Even the role of SGLT-2 on vessels has not been understood yet. It has been shown that chronic treatment of SGLT-2 inhibitors in a diabetic mouse model attenuated vascular relaxation dysfunction and atherosclerosis in aorta and coronary artery [58, 121, 122]. More studies investigating the roles of SGLT-2 inhibitors on the heart are needed to warrant their use in the future.

## Abbreviations

[Ca^2+^]_c_: cytoplasmic Ca^2+^ concentration
[Ca^2+^]_m_: mitochondrial Ca^2+^ concentration
[Na^+^]_c_: cytoplasmic Na^+^ concentration
+ dp/dt: the maximal ascending rate of left ventricular pressure
− dp/dt: the maximal descending rate of left ventricular pressure
AGEs: advanced glycation end products
ASC: apoptosis-associated speck-like protein containing a caspase recruitment domain
ATF4: activating transcription factor 4
CHOP: CCAAT-enhancer-binding protein homologous protein
CPT1: carnitine palmitoyltransferase 1
CVD: cardiovascular diseases
DM: diabetes mellitus
DRP1: dynamin-related protein 1
DT: deceleration time
E wave: mitral inflow peak velocity
E/A: early/late diastolic
EDP: end-diastolic pressure
EDV: end-diastolic volume
EDWT: end diastolic wall thickness
EF: ejection fraction
ENaC: epithelial sodium channel
ER: endoplasmic reticulum
ERS: endoplasmic reticulum stress
ESP: end-systolic pressure
ESV: end-systolic volume
GRP78: glucose-regulated protein 78
HHF: heart failure hospitalization
HR: hazard ratio
I/R: ischemic/reperfusion
IL-1β: interleukin-1β
IVRT: isovolumic relaxation time
LAD: left anterior descending artery
LV: left ventricular
MFN2: mitofusin 2
MI: myocardial infarction
NALP-3: NACHT, LRR and PYD domains-containing protein 3
NCX: Na^+^/Ca^2+^ exchanger
NHE: Na^+^/H^+^ exchange
NLRP3: nucleotide-binding oligomerization domain-like receptor 3
OPA1: optic atrophy 1
PGC1-α: peroxisome proliferator-activated receptor gamma coactivator 1-alpha
RAGEs: receptors for advanced glycation end products
RONS: reactive oxygen and nitrogen species
ROS: reactive oxygen species
RRR: relative risk reduction
SERCA2a: sarcoplasmic endoplasmic reticulum Ca^2+^-ATPase
SGK1: serum- and glucocorticoid-regulated kinase 1
SGLT: sodium–glucose co-transporters
STAT3: signal transducer and activator of the transcription 3
T2DM: type 2 diabetes mellitus
Tau: time constant for isovolumic relaxation
TRAF2: tumor necrosis factor receptor-associated factor 2
XBP1: X-box binding protein 1
